# Prevalence and genotypic distribution of non-epidermolytic ichthyosis in Italian Golden Retrievers

**DOI:** 10.1371/journal.pone.0345595

**Published:** 2026-03-24

**Authors:** Maria Grazia De Iorio, Michele Polli, Sara Ghilardi, Stefano Frattini, Mara Bagardi, Alessandra Paganelli, Maria Cristina Cozzi, Kenza Seghrouchni, Paola Giuseppina Brambilla, Giulietta Minozzi

**Affiliations:** 1 Department of Veterinary Medicine and Animal Science - DIVAS, University of Milan, Lodi, Italy; 2 Vetogene Laboratory—ENCI Servizi SRL, Milan, Italy; 3 Department of Veterinary Medicine and Animal Sciences, University of Messina, Messina, Italy; The Islamia University of Bahawalpur, PAKISTAN

## Abstract

Non-epidermolytic ichthyosis (NEI) is a hereditary skin disorder affecting several dog breeds, most notably the Golden Retriever. It is primarily caused by a loss-of-function variant in the *PNPLA1* gene, while a second, less common form is associated with a deletion in the *ABHD5* gene. This retrospective study aimed to assess the prevalence and temporal trends of both mutations in Golden Retrievers tested in Italy between 2017 and September 2025. A total of 508 genetic tests were analyzed, including 463 dogs tested for the *PNPLA1* mutation, 42 for the *ABHD5* deletion, and 3 for both variants. DNA was extracted from blood or buccal samples and analyzed by real-time PCR followed by confirmatory Sanger sequencing. Among the *PNPLA1* tested dogs, 42% were clears (wt/wt), 37% carriers (wt/mut), and 21% affected (mut/mut), with calculated allele frequencies of 60% wild-type and 40% mutant. A significant temporal decline in mutant allele frequency was observed, accompanied by an increasing number of animals tested over time, suggesting growing interest in genetic screening and its impact on selective breeding. Conversely, all dogs tested for the *ABHD5* deletion were wild-type, supporting its rarity in the breed. Overall, these findings confirm that *PNPLA1*-related ichthyosis remains one of the most prevalent hereditary disorders in Golden Retrievers, although its frequency is decreasing. The results emphasize the effectiveness of genetic testing in disease prevention and highlight the importance of continued monitoring to maintain genetic health within the breed.

## Introduction

Genetic disorders are particularly prevalent in purebred dogs, largely due to the combined effects of artificial selection, reduced effective population size, and increased inbreeding coefficients [1–4]. Indeed, selective breeding has often prioritized morphological traits over health, leading to the fixation of deleterious alleles and a consequent rise in breed-specific hereditary conditions [2–6]. Among these, genodermatoses, monogenic hereditary skin disorders, are of particular concern. Dogs, second only to humans, present the highest number of identified inherited diseases, many of which affect the skin, such as Ichthyosis [7–9]. Ichthyosis is a rare congenital or hereditary skin disease caused by primary defects in the formation of the stratum corneum [7]. It is classified into epidermolytic and non-epidermolytic forms, depending on the presence or absence of keratinocyte vacuolization and lysis in the spinous and granular layers [10,11]. Epidermolytic ichthyosis has been reported in breeds such as the Norfolk Terrier due to mutations in keratin genes [12], while non- epidermolytic ichthyosis (NEI) is more common and has been described in Golden Retrievers, Jack Russell Terriers, American Bulldogs, Shar-Pey and German shepherd [8,13,14].

In Golden Retrievers, NEI is most commonly associated with an insertion–deletion variant in exon 8 of *patatin-like phospholipase domain containing 1* (*PNPLA1*) gene, located on chromosome 11 (NC_051816.1: 5,725,170–5,771,521) [15,16]. More recently, a second form of ichthyosis, referred to as Golden Retriever ichthyosis type 2, has been described and associated with a 14 bp deletion in the *ABHD5* gene (c.1006_1019del), although its occurrence appears to be much rarer within the breed [17]. The *PNPLA1* gene encodes a patatin-like phospholipase, while *ABHD5* encodes the α/β-hydrolase domain-containing protein 5; both proteins are involved in epidermal lipid metabolism, particularly in the biosynthesis of ω-O-acylceramides, which are essential for skin barrier integrity [18–23]. Loss-of-function variants in *PNPLA1* or *ABHD5* have been linked to autosomal recessive congenital ichthyosis in both dogs and humans [16,19].

In both mutations, affected Golden Retrievers usually develop clinical signs early in life, characterized by generalized scaling, hyperpigmented and rough skin in ventral glabrous areas, and laminated orthokeratosis on histopathology [10,13]. The condition is generally non-pruritic, although secondary infections may cause inflammation and discomfort [13].

Given the hereditary nature and the high prevalence of NEI, genetic testing is a key preventive tool [24,25]. It enables the identification of carriers and affected dogs guiding breeding strategies to reduce mutation prevalence while maintaining genetic diversity.

The aim of this study was to assess the prevalence of the two mutations associated with non-epidermolytic ichthyosis (NEI) in Golden Retrievers in Italy.

## Materials and methods

### Sampling

This retrospective observational study included DNA test results for two genetic variants associated with non-epidermolytic ichthyosis (NEI) in Golden Retrievers, analyzed between 2017 and September 2025 in Italy by the Vetogene Laboratory.

A total of 508 tests were included: 460 dogs were tested only for the *PNPLA1* gene mutation (type 1 NEI), 42 only for the *ABHD5* gene deletion (type 2 NEI), and three for both variants. Screening for the *PNPLA1* mutation was performed throughout the entire study period, whereas testing for the *ABHD5* deletion has been available only since 2024.

All data were obtained from routine genetic testing requested by owners, breeders, or veterinarians, primarily for breeding management or diagnostic purposes. For this reason, ethical approval was not required, while informed owner consent was obtained for the use of genetic results for research purposes.

Individual-level data for all tested dogs, including genotype results and demographic variables, are provided in the Supplementary Material (S1 and S2 Tables).

### DNA genotyping

Biological samples consisted of EDTA blood, buccal swabs (GenoTube®), or Vetkard® blood cards, collected by veterinarians following standard procedures. DNA extraction was performed at the Vetogene Laboratory using commercial kits (E.Z.N.A.® Blood DNA Purification Kit, Omega Bio-tek, Norcross, GA, USA) according to the manufacturer’s instructions, with minor adaptations depending on sample type. After extraction, DNA samples were sent to EuroVetGene Molecular Diagnostics, an accredited commercial laboratory, where real-time PCR analyses were performed to identify the genotype based on melting curve profiles. The analytical protocol was identical for both variants, with the specific mutation (*PNPLA1* or *ABHD5*) tested according to the owner’s request. Genotyping results were then confirmed by direct sequencing using the Sanger method to validate the findings [16,24]. Based on these analyses, dogs were classified as clears (homozygous wt/wt), carriers (heterozygous wt/mut) and affected (homozygous mut/mut).

### Statistical analyses

Statistical analyses were conducted using R software (R Core Team). Differences in genotype frequencies among groups were first assessed using the Chi-square test with the “chisq.test” function in R applied to global contingency tables, in order to evaluate overall associations between grouping variables and genotype frequencies. A p-value < 0.05 was considered statistically significant. When a significant overall difference were detected, pairwise post-hoc comparisons were carried out using Fisher’s exact test between each pair of groups, to account for small sample sizes and sparse contingency tables. Allele frequencies for the wild-type (wt) and mutant (mut) variants were calculated according to the following formulas:


$$\text{f}(\text{wt})=\frac{\text{2Nwtwt }+\text{ Nwtmut}}{\text{2N}}$$ (1)

$$\text{f}(\text{mut})=\frac{\text{2Nmutmut}+\text{ Nwtmut}}{\text{2N}}$$ (2)

## Results

Among the 463 Golden Retrievers tested for *PNPLA1* mutation between 2017 and September 2025, 42% (n = 192) were classified as clears (wt/wt), 37% (n = 173) as carriers (wt/mut), and 21% (n = 98) as affected (mut/mut). The calculated allele frequencies were 60% for the wild-type allele and 40% for the mutant allele, indicating an almost balanced distribution of the two variants in the analyzed population.

In addition, 45 dogs were tested for the *ABHD5* gene deletion (type 2 NEI), including two in 2024 and 43 in 2025. All individuals were clears, resulting in a wild-type allele frequency of 100%.

Of the three dogs tested for both genes, one was affected, one a carrier, and one clear for the *PNPLA1* mutation, while all three were wild-type for *ABHD5*.

### Sex and age distribution of *PNPLA1* genotypes

Of all dogs tested for *PNPLA1*, 162 were males (35%) and 301 were females (65%). The genotype distribution was overall comparable between sexes (S3 Table). The higher number of females tested likely reflects breeding management practices or population structure, as females typically represent a larger share of the reproductive population.

When grouped by age, the majority of tests were performed on dogs between 0 and 4 years old, with a peak at two years (n = 137) (S4 Table). This indicates that genetic testing is usually performed in young animals, often before or around the onset of the reproductive period. A smaller proportion of tests involved older dogs, likely reflecting diagnostic screening following the appearance of clinical signs rather than preventive testing. No statistically significant difference in genotype distribution was observed among age groups (p > 0.05), confirming that the apparent variations are related to sampling behavior rather than biological effects.

### Temporal distribution of *PNPLA1* genotypes

The number of samples tested for *PNPLA1* varied over the years, showing an overall increasing trend despite some fluctuations (Table 1). The number of analyzed dogs progressively rose from 2 in 2017 to 126 in 2025, reaching the highest value even though the dataset includes data only up to September. Except for 2017 (n = 2), which is too small to be representative of the genotypic frequencies, the early years of sampling showed relatively high proportions of affected dogs, peaking at 44% in 2020. In the following years, this percentage gradually declined, reaching 14% in 2025. The proportion of carriers remained stable, with minor annual fluctuation. Overall, these results indicate both a progressive reduction in the frequency of affected individuals and an increase in the proportion of clear dogs, alongside a steady rise in the total number of animals tested over time. Statistical analysis confirmed a significant difference in genotype distribution across years (p = 0.01). Post-hoc comparisons revealed that this significance was mainly driven by differences between 2020 vs. 2025 (p = 0.00003), 2020 vs. 2024 (p = 0.0008), and 2020 vs. 2023 (p = 0.003).

**Table 1:** Annual distribution and frequencies of *PNPLA1* genotypes.

Year of sampling	N° of samples	Clears		Affected		Carriers	
		N°	Freq	N°	Freq	N°	Freq
2017	2	2	100%	0	0%	0	0%
2018	11	3	27%	3	27%	5	45%
2019	12	4	33%	4	33%	4	33%
2020	48	10	21%	21	44%	17	35%
2021	72	25	35%	18	25%	29	40%
2022	36	13	36%	8	22%	15	42%
2023	45	22	49%	7	16%	16	36%
2024	111	48	43%	19	17%	44	40%
2025	126	65	52%	18	14%	43	34%

N° = Number of samples.

Freq = Frequencies.

**Fig 1:**
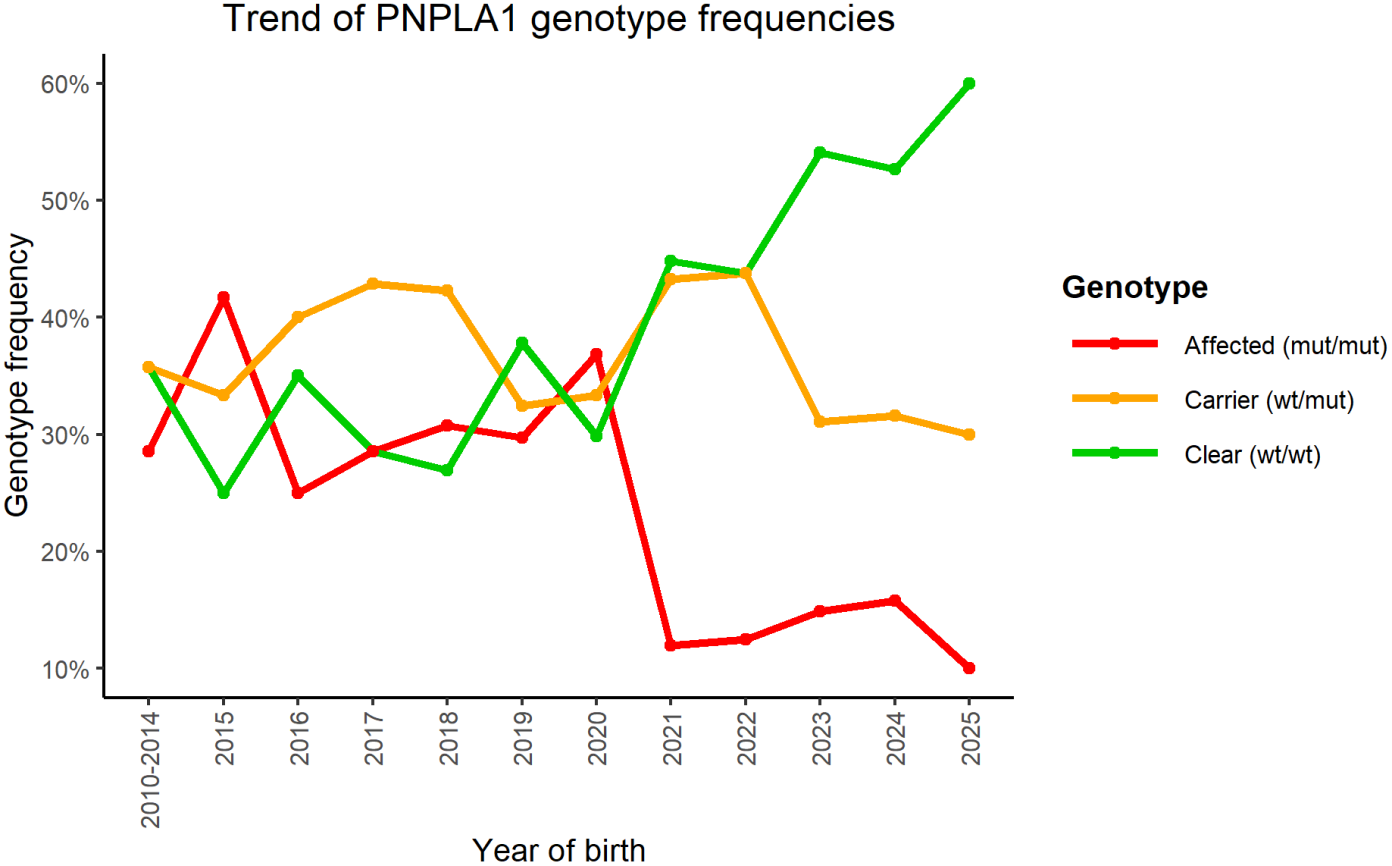
Temporal trend of *PNPLA1* genotypic frequencies by year of birth in tested Golden Retrievers from 2015 to 2025. The graph shows the frequency of clear (wt/wt) in green, carriers (wt/mut) in orange, and affected (mut/mut) in red.

When considering the year of birth of the tested dogs instead of the year of sampling, a more accurate representation of the population trend emerges (Table 2). When genotypes were analyzed according to year of birth, a significant difference in genotype distribution was detected (p = 0.04). Dogs born in earlier years showed higher proportions of affected individuals, whereas more recent birth cohorts (2021–2025) exhibited lower frequencies of affected and higher proportions of clears, culminating in 2025-born dogs with only 10% affected and 60% clear individuals (Fig 1).

**Table 2:** *PNPLA1* genotype frequencies by year of birth.

Year of birth	N° of samples	Clear		Affected		Carriers	
		N°	Freq	N°	Freq	N°	Freq
2025	10	6	60%	1	10%	3	30%
2024	38	20	53%	6	16%	12	32%
2023	74	40	54%	11	15%	23	31%
2022	80	35	44%	10	13%	35	44%
2021	67	30	45%	8	12%	29	43%
2020	57	17	30%	21	37%	19	33%
2019	37	14	38%	11	30%	12	32%
2018	26	7	27%	8	31%	11	42%
2017	28	8	29%	8	29%	12	43%
2016	20	7	35%	5	25%	8	40%
2015	12	3	25%	5	42%	4	33%
2010-2014	14	5	36%	4	29%	5	36%

N° = Number of samples

Freq = Frequencies

Post-hoc pairwise comparisons indicated that the significance was driven by differences between 2020 vs. 2022 (p = 0.003), 2020 vs. 2021 (p = 0.005), 2020 vs. 2023 (p = 0.005), 2020 vs. 2024 (p = 0.04), and 2018 vs. 2023 (p = 0.04).

## Discussion

Non-epidermolytic ichthyosis (NEI) in Golden Retrievers is a hereditary skin disorder caused by a loss-of-function insertion–deletion variant in the *PNPLA1* gene, which plays a key role in epidermal lipid metabolism and skin barrier formation [16,18–20]. More recently, a second form, linked to a 14 bp deletion in the *ABHD5* gene, has been identified, although its occurrence appears to be much rarer within the breed [17]. Both variants result in clinically similar non-epidermolytic ichthyosis phenotypes.

Given the much higher number of samples analyzed for *PNPLA1*, this study primarily focuses on this mutation, which remains the major genetic cause of NEI in Golden Retrievers and the main target of screening programs.

In our study, 463 Golden Retrievers were tested in Italy between 2017 and September 2025 for the *PNPLA1* mutation. The results showed a predominance of the wild-type genotype compared to previous reports, with 42% clears, 37% carriers, and 21% affected. The calculated allele frequencies were 60% for the wild-type allele and 40% for the mutant allele, indicating a moderate reduction in the prevalence of the mutation compared with previous reports.

For instance, Owczarek-Lipska et al. [25] in Switzerland found 20% clear, 49% carriers, and 32% affected, with a mutant allele frequency of 56.1%, slightly higher than our estimate. Similarly, Roething et al. [26] in Germany reported mutant allele frequencies of 54.5% in puppies and 58.8% in dams. By contrast, a multi-country study [27] highlighted marked geographical differences. The mutant allele frequencies were found lower in Australia (31.5%) and in the USA (38.5%), the latter closely aligning with our estimate (40%). In contrast European values were consistently higher, ranging from 48.5% in French samples to 64.5% in Swiss (Guaguère et al., 2013).

The consistently high prevalence of the *PNPLA1* mutation reported across different countries suggests that its distribution within the Golden Retriever breed may be compatible with a founder effect, potentially amplified by the widespread use of popular sires and the restricted genetic diversity typical of purebred dog populations [1–4]. However, dedicated population genetic or pedigree-based analyses would be required to formally test this hypothesis.

A comparison with another Italian study based on 48 samples conducted in 2018 [24] reveals a higher prevalence of the mutant allele. In that work, carriers represented the largest group (48%) and the proportion of affected dogs was also higher (31%) than in our cohort (21%), resulting in a mutant allele frequency of 55.2%, markedly above the 40% observed in our dataset. Both studies also examined genotype distribution by sex; however, the strong imbalance reported by Graziano et al. [24], with males more frequently clears (30%) and females predominantly affected (46%), was not confirmed in our population (S3 Table).

When considering only the dogs born before 2018, our results align closely with those reported by Graziano et al. in 2018 [24], showing a mutant allele frequency of 50%, with 30% of affected individuals. These higher percentages are also consistent with those described in previous European studies, reinforcing the idea that the differences between our findings and earlier reports are mainly attributable to the time period covered.

Indeed, our data show a statistically significant temporal shift in genotype distribution, both when analyzed by year of sampling and by year of birth. As illustrated in Fig 1, since 2020 there has been a marked increase in the proportion of clear dogs and a concurrent decrease in affected individuals, while carriers have remained relatively stable despite minor annual fluctuations. This pattern is consistent with the increasing adoption of genetic screening and selective breeding strategies aimed at reducing the prevalence of the *PNPLA1* mutation. However, the dataset is based on voluntary genetic testing requested mainly for breeding or diagnostic purposes and therefore does not represent a random sample of the Italian Golden Retriever population. Consequently, the observed temporal decline in mutant allele frequency may partly reflect changes in testing practices and selection strategies rather than being solely attributable to a reduction in the frequency of the *PNPLA1* mutation.

It is also worth noting that the majority of the dogs were tested at a young age, with 64% of samples collected from individuals aged 0–2 years. This further supports the increasing focus on early genetic screening, reflecting breeders’ attention to preventive selection practices within the population.

However, despite the evident decreasing trend, the incidence of the mutation remains high, with an overall mutant allele frequency of 40%. This value is very high compared with other hereditary mutations investigated in Italian dog populations, which usually range between 6% and 10% [28–31]. When considering other hereditary conditions reported in Italian Golden Retrievers, such as progressive retinal atrophy, the prevalence was estimated at 4.2% for PRA1 and 6.5% for PRA2 [29], values markedly lower than those observed for ichthyosis. These results confirm that *PNPLA1*-related ichthyosis is among the most prevalent and clinically relevant inherited disorders in the breed, consistent with Donner et al. [1], who ranked it among the 20 most common hereditary diseases in both purebred and mixed-breed dogs.

Conversely, for the *ABHD5* gene deletion (type 2 NEI), all 45 dogs tested were clears, resulting in a wild-type allele frequency of 100%. These dogs, evenly distributed between males (n = 20) and females (n = 25) and born between 2020 and 2024, represent the first data available for this recently identified mutation, described only in 2022 by Kiener et al. [17]. Given the recent introduction of *ABHD5* testing and the limited sample size, these findings apply exclusively to the tested population and do not allow conclusions about the overall prevalence of this variant in the breed. To date, *ABHD5*-related ichthyosis has been reported only in a limited number of cases, and additional data will be required to better define its distribution within Golden Retrievers. Nevertheless, continued genetic testing for *ABHD5* is advisable, as early monitoring of this variant could help prevent its spread in the population should it emerge in the future.

Moreover, as observed for *PNPLA1*, *ABHD5* testing also showed increasing attention, with the number of samples rising from only two in 2024–43 in 2025.

## Conclusions

This study provides an updated overview of non-epidermolytic ichthyosis in Golden Retrievers in Italy, focusing on both the *PNPLA1* and *ABHD5* variants. The results confirm that *PNPLA1*-related ichthyosis remains widespread, with a mutant allele frequency of 40%, although lower than that reported in previous Italian and European studies. The progressive decrease in affected and carrier dogs observed over recent years, together with the marked rise in the number of genetic tests performed, reflects growing awareness and the effectiveness of preventive breeding strategies, although changes in testing behavior and selective screening may also contribute to the observed reduction in the mutation frequency.

In contrast, all dogs tested for the *ABHD5* deletion were clears, indicating that this recently described mutation was not detected in the tested population. However, given the limited and recent dataset, conclusions regarding the overall prevalence of *ABHD5*-related ichthyosis in the breed cannot be drawn. Continued surveillance and early genetic testing for both variants are essential to prevent the spread of deleterious alleles and to promote the long-term genetic health of the breed.

## Supporting information

S1 Table Individual-level data for PNPLA1 genotyping in Italian Golden Retrievers. The table includes genotype classification (clear, carrier, affected), sex, year of birth, year of sampling, and age at sampling for all 463 tested dogs.
(DOCX)

S2 Table Individual-level data for ABHD5 genotyping in Italian Golden Retrievers. The table includes ABHD5 genotype results, PNPLA1 genotype (when available), sex, year of birth, year of sampling, and age at sampling for all tested dogs.
(DOCX)

S3 Table *PNPLA1* genotype frequencies by sex. The table reports the number and frequency of dogs classified as clear, affected, and carrier, stratified by sex.
(DOCX)

S4 Table *PNPLA1* genotype frequencies by age. The table reports the number and frequency of dogs classified as clear, affected, and carrier, stratified by age class.
(DOCX)
