# Supplementary material for: Prevalence and genotypic distribution of non-epidermolytic ichthyosis in Italian Golden Retrievers
Source: PLoS One. 2026 Mar 24;21(3):e0345595. doi: 10.1371/journal.pone.0345595 (PMC13012511; doi:10.1371/journal.pone.0345595)
Supplement: S1 Table — The table includes genotype classification (clear, carrier, affected), sex, year of birth, year of sampling, and age at sampling for all 463 tested dogs. (DOCX) [file pone.0345595.s001.docx]

**S1 Table. Individual-level data for PNPLA1 genotyping in Italian Golden Retrievers.** The table includes genotype classification (clear, carrier, affected), sex, year of birth, year of sampling, and age at sampling for all 463 tested dogs.

| **ID** | **PNPLA1** | **Sex** | **Year of birth** | **Year of Sampling** | **Age** |
| --- | --- | --- | --- | --- | --- |
| 1 | CLEAR | M | 2010 | 2017 | 7 |
| 2 | CLEAR | M | 2015 | 2017 | 2 |
| 3 | CARRIER | M | 2013 | 2018 | 5 |
| 4 | CARRIER | F | 2015 | 2018 | 3 |
| 5 | AFFECTED | F | 2015 | 2018 | 3 |
| 6 | CLEAR | F | 2016 | 2018 | 2 |
| 7 | CLEAR | M | 2014 | 2018 | 4 |
| 8 | CARRIER | M | 2015 | 2018 | 4 |
| 9 | AFFECTED | M | 2014 | 2018 | 4 |
| 10 | CARRIER | F | 2016 | 2018 | 2 |
| 11 | AFFECTED | F | 2017 | 2018 | 1 |
| 12 | CARRIER | F | 2018 | 2018 | 0 |
| 13 | CLEAR | M | 2016 | 2018 | 2 |
| 14 | CLEAR | M | 2011 | 2019 | 8 |
| 15 | CARRIER | M | 2014 | 2019 | 5 |
| 16 | CARRIER | F | 2014 | 2019 | 5 |
| 17 | CARRIER | F | 2015 | 2019 | 4 |
| 18 | CLEAR | F | 2016 | 2019 | 3 |
| 19 | AFFECTED | F | 2017 | 2019 | 2 |
| 20 | CARRIER | F | 2018 | 2019 | 1 |
| 21 | AFFECTED | M | 2015 | 2019 | 4 |
| 22 | CLEAR | F | 2019 | 2019 | 0 |
| 23 | AFFECTED | M | 2017 | 2019 | 2 |
| 24 | AFFECTED | M | 2017 | 2019 | 2 |
| 25 | CLEAR | M | 2018 | 2019 | 1 |
| 26 | AFFECTED | M | 2016 | 2020 | 4 |
| 27 | CARRIER | F | 2014 | 2020 | 6 |
| 28 | CLEAR | F | 2015 | 2020 | 5 |
| 29 | CARRIER | F | 2015 | 2020 | 5 |
| 30 | AFFECTED | M | 2016 | 2020 | 4 |
| 31 | AFFECTED | F | 2015 | 2020 | 5 |
| 32 | AFFECTED | F | 2016 | 2020 | 4 |
| 33 | CARRIER | F | 2016 | 2020 | 4 |
| 34 | CARRIER | F | 2016 | 2020 | 4 |
| 35 | AFFECTED | F | 2016 | 2020 | 4 |
| 36 | CARRIER | F | 2017 | 2020 | 3 |
| 37 | AFFECTED | F | 2017 | 2020 | 3 |
| 38 | CLEAR | F | 2017 | 2020 | 3 |
| 39 | CLEAR | F | 2018 | 2020 | 2 |
| 40 | AFFECTED | F | 2018 | 2020 | 2 |
| 41 | AFFECTED | M | 2017 | 2020 | 3 |
| 42 | CARRIER | F | 2018 | 2020 | 2 |
| 43 | AFFECTED | F | 2018 | 2020 | 2 |
| 44 | CARRIER | F | 2018 | 2020 | 2 |
| 45 | AFFECTED | F | 2018 | 2020 | 2 |
| 46 | CARRIER | F | 2018 | 2020 | 2 |
| 47 | AFFECTED | F | 2018 | 2020 | 2 |
| 48 | CARRIER | F | 2018 | 2020 | 2 |
| 49 | CARRIER | F | 2019 | 2020 | 1 |
| 50 | CARRIER | F | 2019 | 2020 | 1 |
| 51 | CLEAR | F | 2019 | 2020 | 1 |
| 52 | CLEAR | F | 2019 | 2020 | 1 |
| 53 | AFFECTED | F | 2019 | 2020 | 1 |
| 54 | CARRIER | F | 2019 | 2020 | 1 |
| 55 | AFFECTED | F | 2020 | 2020 | 0 |
| 56 | CLEAR | M | 2017 | 2020 | 3 |
| 57 | AFFECTED | F | 2020 | 2020 | 0 |
| 58 | AFFECTED | F | 2020 | 2020 | 0 |
| 59 | CLEAR | M | 2018 | 2020 | 2 |
| 60 | AFFECTED | F | 2020 | 2020 | 0 |
| 61 | CLEAR | M | 2018 | 2020 | 2 |
| 62 | CARRIER | M | 2018 | 2020 | 2 |
| 63 | CARRIER | M | 2018 | 2020 | 2 |
| 64 | AFFECTED | M | 2019 | 2020 | 1 |
| 65 | CARRIER | M | 2019 | 2020 | 1 |
| 66 | CARRIER | M | 2019 | 2020 | 1 |
| 67 | CLEAR | M | 2020 | 2020 | 0 |
| 68 | CARRIER | M | 2020 | 2020 | 0 |
| 69 | CLEAR | M | 2020 | 2020 | 0 |
| 70 | AFFECTED | M | 2020 | 2020 | 0 |
| 71 | AFFECTED | M | 2020 | 2020 | 0 |
| 72 | AFFECTED | M | 2020 | 2020 | 0 |
| 73 | AFFECTED | M | 2020 | 2020 | 0 |
| 74 | AFFECTED | M | 2013 | 2021 | 8 |
| 75 | CLEAR | M | 2013 | 2021 | 8 |
| 76 | AFFECTED | M | 2015 | 2021 | 6 |
| 77 | CLEAR | M | 2016 | 2021 | 5 |
| 78 | CLEAR | M | 2017 | 2021 | 4 |
| 79 | CARRIER | M | 2017 | 2021 | 4 |
| 80 | CARRIER | M | 2017 | 2021 | 4 |
| 81 | CLEAR | M | 2017 | 2021 | 4 |
| 82 | CARRIER | M | 2017 | 2021 | 4 |
| 83 | AFFECTED | F | 2015 | 2021 | 6 |
| 84 | CLEAR | F | 2015 | 2021 | 6 |
| 85 | CARRIER | F | 2016 | 2021 | 5 |
| 86 | AFFECTED | F | 2016 | 2021 | 5 |
| 87 | CARRIER | F | 2016 | 2021 | 5 |
| 88 | CLEAR | F | 2016 | 2021 | 5 |
| 89 | CLEAR | F | 2016 | 2021 | 5 |
| 90 | CARRIER | F | 2016 | 2021 | 5 |
| 91 | CARRIER | F | 2017 | 2021 | 4 |
| 92 | CLEAR | F | 2017 | 2021 | 4 |
| 93 | AFFECTED | F | 2017 | 2021 | 4 |
| 94 | CARRIER | F | 2017 | 2021 | 4 |
| 95 | CLEAR | F | 2017 | 2021 | 4 |
| 96 | CARRIER | F | 2017 | 2021 | 4 |
| 97 | CLEAR | F | 2017 | 2021 | 4 |
| 98 | CARRIER | F | 2017 | 2021 | 4 |
| 99 | CARRIER | F | 2017 | 2021 | 4 |
| 100 | AFFECTED | F | 2018 | 2021 | 3 |
| 101 | AFFECTED | F | 2018 | 2021 | 3 |
| 102 | AFFECTED | F | 2018 | 2021 | 3 |
| 103 | CARRIER | F | 2018 | 2021 | 3 |
| 104 | CLEAR | F | 2018 | 2021 | 3 |
| 105 | CARRIER | F | 2018 | 2021 | 3 |
| 106 | AFFECTED | F | 2019 | 2021 | 2 |
| 107 | CLEAR | F | 2019 | 2021 | 2 |
| 108 | CLEAR | F | 2019 | 2021 | 2 |
| 109 | CARRIER | F | 2019 | 2021 | 2 |
| 110 | AFFECTED | F | 2019 | 2021 | 2 |
| 111 | CLEAR | M | 2019 | 2021 | 2 |
| 112 | CARRIER | F | 2019 | 2021 | 2 |
| 113 | CLEAR | F | 2019 | 2021 | 2 |
| 114 | AFFECTED | F | 2019 | 2021 | 2 |
| 115 | CLEAR | F | 2019 | 2021 | 2 |
| 116 | CARRIER | F | 2019 | 2021 | 2 |
| 117 | CARRIER | F | 2019 | 2021 | 2 |
| 118 | CLEAR | F | 2019 | 2021 | 2 |
| 119 | CLEAR | F | 2019 | 2021 | 2 |
| 120 | AFFECTED | F | 2019 | 2021 | 2 |
| 121 | AFFECTED | F | 2020 | 2021 | 1 |
| 122 | CLEAR | F | 2020 | 2021 | 1 |
| 123 | CARRIER | F | 2020 | 2021 | 1 |
| 124 | CLEAR | F | 2020 | 2021 | 1 |
| 125 | CARRIER | F | 2020 | 2021 | 1 |
| 126 | AFFECTED | F | 2020 | 2021 | 1 |
| 127 | AFFECTED | F | 2020 | 2021 | 1 |
| 128 | AFFECTED | F | 2020 | 2021 | 1 |
| 129 | CARRIER | M | 2019 | 2021 | 2 |
| 130 | CARRIER | F | 2020 | 2021 | 1 |
| 131 | CARRIER | F | 2020 | 2021 | 1 |
| 132 | CARRIER | F | 2020 | 2021 | 1 |
| 133 | CARRIER | M | 2020 | 2021 | 1 |
| 134 | CARRIER | F | 2021 | 2021 | 0 |
| 135 | CARRIER | F | 2021 | 2021 | 0 |
| 136 | CLEAR | F | 2021 | 2021 | 0 |
| 137 | AFFECTED | F | 2021 | 2021 | 0 |
| 138 | CARRIER | F | 2021 | 2021 | 0 |
| 139 | CARRIER | F | 2021 | 2021 | 0 |
| 140 | CLEAR | M | 2020 | 2021 | 1 |
| 141 | AFFECTED | M | 2021 | 2021 | 0 |
| 142 | CARRIER | M | 2021 | 2021 | 0 |
| 143 | CLEAR | M | 2021 | 2021 | 0 |
| 144 | CLEAR | M | 2021 | 2021 | 0 |
| 145 | CLEAR | M | 2021 | 2021 | 0 |
| 146 | AFFECTED | M | 2014 | 2022 | 8 |
| 147 | CARRIER | M | 2016 | 2022 | 6 |
| 148 | CARRIER | M | 2017 | 2022 | 5 |
| 149 | AFFECTED | M | 2018 | 2022 | 4 |
| 150 | CLEAR | M | 2018 | 2022 | 4 |
| 151 | CLEAR | M | 2019 | 2022 | 3 |
| 152 | AFFECTED | F | 2014 | 2022 | 8 |
| 153 | CLEAR | F | 2019 | 2022 | 3 |
| 154 | CARRIER | F | 2020 | 2022 | 2 |
| 155 | CARRIER | F | 2020 | 2022 | 2 |
| 156 | AFFECTED | F | 2020 | 2022 | 2 |
| 157 | CLEAR | M | 2020 | 2022 | 2 |
| 158 | CLEAR | F | 2020 | 2022 | 2 |
| 159 | CLEAR | F | 2020 | 2022 | 2 |
| 160 | CARRIER | M | 2020 | 2022 | 2 |
| 161 | CLEAR | F | 2020 | 2022 | 2 |
| 162 | AFFECTED | F | 2020 | 2022 | 2 |
| 163 | AFFECTED | F | 2020 | 2022 | 2 |
| 164 | CARRIER | F | 2020 | 2022 | 2 |
| 165 | CARRIER | F | 2020 | 2022 | 2 |
| 166 | CLEAR | F | 2021 | 2022 | 1 |
| 167 | CARRIER | F | 2021 | 2022 | 1 |
| 168 | CARRIER | F | 2021 | 2022 | 1 |
| 169 | CARRIER | F | 2021 | 2022 | 1 |
| 170 | CARRIER | F | 2021 | 2022 | 1 |
| 171 | CLEAR | F | 2021 | 2022 | 1 |
| 172 | AFFECTED | F | 2021 | 2022 | 1 |
| 173 | CARRIER | F | 2022 | 2022 | 0 |
| 174 | CLEAR | M | 2020 | 2022 | 2 |
| 175 | CLEAR | M | 2020 | 2022 | 2 |
| 176 | CARRIER | M | 2021 | 2022 | 1 |
| 177 | AFFECTED | M | 2021 | 2022 | 1 |
| 178 | CLEAR | M | 2021 | 2022 | 1 |
| 179 | CARRIER | M | 2021 | 2022 | 1 |
| 180 | CLEAR | M | 2021 | 2022 | 1 |
| 181 | CARRIER | M | 2021 | 2022 | 1 |
| 182 | CLEAR | M | 2019 | 2023 | 4 |
| 183 | AFFECTED | M | 2020 | 2023 | 3 |
| 184 | CARRIER | F | 2017 | 2023 | 6 |
| 185 | CLEAR | F | 2017 | 2023 | 6 |
| 186 | CLEAR | F | 2018 | 2023 | 5 |
| 187 | CARRIER | F | 2019 | 2023 | 4 |
| 188 | AFFECTED | F | 2019 | 2023 | 4 |
| 189 | CLEAR | F | 2020 | 2023 | 3 |
| 190 | CARRIER | F | 2020 | 2023 | 3 |
| 191 | AFFECTED | F | 2020 | 2023 | 3 |
| 192 | CLEAR | F | 2020 | 2023 | 3 |
| 193 | CLEAR | F | 2020 | 2023 | 3 |
| 194 | CLEAR | F | 2021 | 2023 | 2 |
| 195 | CLEAR | F | 2021 | 2023 | 2 |
| 196 | CLEAR | F | 2021 | 2023 | 2 |
| 197 | CARRIER | F | 2021 | 2023 | 2 |
| 198 | CARRIER | F | 2021 | 2023 | 2 |
| 199 | CLEAR | F | 2021 | 2023 | 2 |
| 200 | CLEAR | F | 2021 | 2023 | 2 |
| 201 | CLEAR | F | 2021 | 2023 | 2 |
| 202 | CLEAR | F | 2021 | 2023 | 2 |
| 203 | CARRIER | F | 2021 | 2023 | 2 |
| 204 | CARRIER | F | 2021 | 2023 | 2 |
| 205 | AFFECTED | M | 2021 | 2023 | 2 |
| 206 | CARRIER | F | 2022 | 2023 | 1 |
| 207 | CLEAR | F | 2022 | 2023 | 1 |
| 208 | CARRIER | M | 2021 | 2023 | 2 |
| 209 | CARRIER | F | 2022 | 2023 | 1 |
| 210 | CLEAR | M | 2021 | 2023 | 2 |
| 211 | CARRIER | F | 2022 | 2023 | 1 |
| 212 | CLEAR | F | 2022 | 2023 | 1 |
| 213 | AFFECTED | F | 2022 | 2023 | 1 |
| 214 | CARRIER | F | 2022 | 2023 | 1 |
| 215 | CLEAR | F | 2022 | 2023 | 1 |
| 216 | CLEAR | M | 2022 | 2023 | 1 |
| 217 | CARRIER | M | 2022 | 2023 | 1 |
| 218 | CLEAR | M | 2022 | 2023 | 1 |
| 219 | CARRIER | M | 2022 | 2023 | 1 |
| 220 | CARRIER | M | 2022 | 2023 | 1 |
| 221 | CLEAR | M | 2022 | 2023 | 1 |
| 222 | CARRIER | M | 2023 | 2023 | 0 |
| 223 | CLEAR | M | 2023 | 2023 | 0 |
| 224 | AFFECTED | M | 2023 | 2023 | 0 |
| 225 | AFFECTED | M | 2023 | 2023 | 0 |
| 226 | CLEAR | M | 2023 | 2023 | 0 |
| 227 | CARRIER | M | 2016 | 2024 | 8 |
| 228 | CLEAR | M | 2016 | 2024 | 8 |
| 229 | CARRIER | M | 2017 | 2024 | 7 |
| 230 | AFFECTED | M | 2019 | 2024 | 5 |
| 231 | AFFECTED | M | 2020 | 2024 | 4 |
| 232 | CARRIER | M | 2020 | 2024 | 4 |
| 233 | CARRIER | M | 2020 | 2024 | 4 |
| 234 | CLEAR | M | 2021 | 2024 | 3 |
| 235 | AFFECTED | F | 2019 | 2024 | 5 |
| 236 | CARRIER | F | 2019 | 2024 | 5 |
| 237 | AFFECTED | F | 2019 | 2024 | 5 |
| 238 | CARRIER | F | 2020 | 2024 | 4 |
| 239 | CARRIER | F | 2020 | 2024 | 4 |
| 240 | AFFECTED | F | 2020 | 2024 | 4 |
| 241 | CARRIER | F | 2021 | 2024 | 3 |
| 242 | CARRIER | F | 2021 | 2024 | 3 |
| 243 | CARRIER | F | 2021 | 2024 | 3 |
| 244 | CARRIER | F | 2021 | 2024 | 3 |
| 245 | CARRIER | F | 2021 | 2024 | 3 |
| 246 | CARRIER | F | 2021 | 2024 | 3 |
| 247 | CLEAR | F | 2021 | 2024 | 3 |
| 248 | CARRIER | F | 2021 | 2024 | 3 |
| 249 | CLEAR | F | 2021 | 2024 | 3 |
| 250 | CLEAR | F | 2021 | 2024 | 3 |
| 251 | CLEAR | F | 2021 | 2024 | 3 |
| 252 | CLEAR | F | 2021 | 2024 | 3 |
| 253 | CARRIER | F | 2021 | 2024 | 3 |
| 254 | CLEAR | M | 2021 | 2024 | 3 |
| 255 | AFFECTED | F | 2021 | 2024 | 3 |
| 256 | CLEAR | F | 2021 | 2024 | 3 |
| 257 | AFFECTED | F | 2021 | 2024 | 3 |
| 258 | CARRIER | F | 2022 | 2024 | 2 |
| 259 | CARRIER | F | 2022 | 2024 | 2 |
| 260 | CLEAR | F | 2022 | 2024 | 2 |
| 261 | CARRIER | F | 2022 | 2024 | 2 |
| 262 | CLEAR | F | 2022 | 2024 | 2 |
| 263 | AFFECTED | F | 2022 | 2024 | 2 |
| 264 | CARRIER | F | 2022 | 2024 | 2 |
| 265 | CLEAR | M | 2022 | 2024 | 2 |
| 266 | CARRIER | F | 2022 | 2024 | 2 |
| 267 | CLEAR | F | 2022 | 2024 | 2 |
| 268 | CLEAR | F | 2022 | 2024 | 2 |
| 269 | CARRIER | F | 2022 | 2024 | 2 |
| 270 | CARRIER | F | 2022 | 2024 | 2 |
| 271 | CLEAR | F | 2022 | 2024 | 2 |
| 272 | CARRIER | F | 2022 | 2024 | 2 |
| 273 | CLEAR | F | 2022 | 2024 | 2 |
| 274 | CLEAR | F | 2022 | 2024 | 2 |
| 275 | AFFECTED | F | 2022 | 2024 | 2 |
| 276 | CLEAR | F | 2022 | 2024 | 2 |
| 277 | CLEAR | F | 2022 | 2024 | 2 |
| 278 | CLEAR | F | 2022 | 2024 | 2 |
| 279 | CARRIER | F | 2022 | 2024 | 2 |
| 280 | CARRIER | F | 2022 | 2024 | 2 |
| 281 | CLEAR | F | 2022 | 2024 | 2 |
| 282 | CLEAR | F | 2022 | 2024 | 2 |
| 283 | CLEAR | F | 2022 | 2024 | 2 |
| 284 | AFFECTED | F | 2022 | 2024 | 2 |
| 285 | CARRIER | F | 2022 | 2024 | 2 |
| 286 | CARRIER | F | 2023 | 2024 | 1 |
| 287 | CLEAR | F | 2023 | 2024 | 1 |
| 288 | CLEAR | F | 2023 | 2024 | 1 |
| 289 | CARRIER | F | 2023 | 2024 | 1 |
| 290 | CLEAR | F | 2023 | 2024 | 1 |
| 291 | AFFECTED | F | 2023 | 2024 | 1 |
| 292 | CARRIER | F | 2023 | 2024 | 1 |
| 293 | CLEAR | F | 2023 | 2024 | 1 |
| 294 | CARRIER | F | 2023 | 2024 | 1 |
| 295 | CLEAR | F | 2023 | 2024 | 1 |
| 296 | AFFECTED | M | 2022 | 2024 | 2 |
| 297 | CLEAR | F | 2023 | 2024 | 1 |
| 298 | CARRIER | F | 2023 | 2024 | 1 |
| 299 | CLEAR | F | 2023 | 2024 | 1 |
| 300 | CARRIER | F | 2023 | 2024 | 1 |
| 301 | AFFECTED | F | 2023 | 2024 | 1 |
| 302 | CLEAR | M | 2022 | 2024 | 2 |
| 303 | CLEAR | F | 2023 | 2024 | 1 |
| 304 | CLEAR | F | 2023 | 2024 | 1 |
| 305 | CLEAR | F | 2023 | 2024 | 1 |
| 306 | AFFECTED | F | 2023 | 2024 | 1 |
| 307 | CARRIER | F | 2023 | 2024 | 1 |
| 308 | AFFECTED | F | 2023 | 2024 | 1 |
| 309 | CARRIER | F | 2024 | 2024 | 0 |
| 310 | CARRIER | F | 2024 | 2024 | 0 |
| 311 | AFFECTED | F | 2024 | 2024 | 0 |
| 312 | CLEAR | M | 2022 | 2024 | 2 |
| 313 | CARRIER | M | 2022 | 2024 | 2 |
| 314 | CARRIER | F | 2024 | 2024 | 0 |
| 315 | CARRIER | F | 2024 | 2024 | 0 |
| 316 | CLEAR | F | 2024 | 2024 | 0 |
| 317 | CARRIER | F | 2024 | 2024 | 0 |
| 318 | CLEAR | F | 2024 | 2024 | 0 |
| 319 | CLEAR | F | 2024 | 2024 | 0 |
| 320 | CLEAR | F | 2024 | 2024 | 0 |
| 321 | CLEAR | M | 2022 | 2024 | 2 |
| 322 | CARRIER | M | 2022 | 2024 | 2 |
| 323 | AFFECTED | M | 2023 | 2024 | 1 |
| 324 | CLEAR | M | 2023 | 2024 | 1 |
| 325 | CARRIER | M | 2023 | 2024 | 1 |
| 326 | CLEAR | M | 2023 | 2024 | 1 |
| 327 | CLEAR | M | 2023 | 2024 | 1 |
| 328 | CLEAR | M | 2023 | 2024 | 1 |
| 329 | CLEAR | M | 2023 | 2024 | 1 |
| 330 | CARRIER | M | 2023 | 2024 | 1 |
| 331 | CLEAR | M | 2023 | 2024 | 1 |
| 332 | CLEAR | M | 2024 | 2024 | 0 |
| 333 | AFFECTED | M | 2024 | 2024 | 0 |
| 334 | CARRIER | M | 2024 | 2024 | 0 |
| 335 | CLEAR | M | 2024 | 2024 | 0 |
| 336 | CARRIER | M | 2024 | 2024 | 0 |
| 337 | AFFECTED | M | 2024 | 2024 | 0 |
| 338 | CARRIER | M | 2015 | 2025 | 10 |
| 339 | AFFECTED | M | 2017 | 2025 | 8 |
| 340 | AFFECTED | M | 2019 | 2025 | 6 |
| 341 | CLEAR | M | 2019 | 2025 | 6 |
| 342 | CARRIER | M | 2020 | 2025 | 5 |
| 343 | CARRIER | M | 2020 | 2025 | 5 |
| 344 | CARRIER | M | 2021 | 2025 | 4 |
| 345 | CARRIER | M | 2021 | 2025 | 4 |
| 346 | AFFECTED | M | 2021 | 2025 | 4 |
| 347 | CARRIER | M | 2022 | 2025 | 3 |
| 348 | AFFECTED | M | 2022 | 2025 | 3 |
| 349 | CLEAR | M | 2022 | 2025 | 3 |
| 350 | CARRIER | M | 2022 | 2025 | 3 |
| 351 | AFFECTED | M | 2022 | 2025 | 3 |
| 352 | CLEAR | M | 2022 | 2025 | 3 |
| 353 | AFFECTED | M | 2022 | 2025 | 3 |
| 354 | CARRIER | M | 2022 | 2025 | 3 |
| 355 | CLEAR | F | 2014 | 2025 | 11 |
| 356 | CARRIER | F | 2018 | 2025 | 7 |
| 357 | AFFECTED | F | 2020 | 2025 | 5 |
| 358 | CARRIER | M | 2022 | 2025 | 3 |
| 359 | CLEAR | F | 2020 | 2025 | 5 |
| 360 | AFFECTED | F | 2020 | 2025 | 5 |
| 361 | CLEAR | F | 2020 | 2025 | 5 |
| 362 | CLEAR | F | 2020 | 2025 | 5 |
| 363 | CLEAR | F | 2021 | 2025 | 3 |
| 364 | CLEAR | F | 2021 | 2025 | 4 |
| 365 | CLEAR | M | 2023 | 2025 | 2 |
| 366 | CLEAR | F | 2021 | 2025 | 4 |
| 367 | CARRIER | F | 2021 | 2025 | 4 |
| 368 | CLEAR | F | 2021 | 2025 | 4 |
| 369 | CLEAR | F | 2021 | 2025 | 4 |
| 370 | CLEAR | F | 2021 | 2025 | 3 |
| 371 | CARRIER | F | 2021 | 2025 | 4 |
| 372 | CLEAR | F | 2022 | 2025 | 3 |
| 373 | AFFECTED | F | 2022 | 2025 | 3 |
| 374 | CLEAR | F | 2022 | 2025 | 3 |
| 375 | AFFECTED | F | 2022 | 2025 | 3 |
| 376 | CLEAR | F | 2022 | 2025 | 3 |
| 377 | CARRIER | F | 2022 | 2025 | 3 |
| 378 | CLEAR | F | 2022 | 2025 | 3 |
| 379 | CLEAR | M | 2023 | 2025 | 2 |
| 380 | CARRIER | F | 2022 | 2025 | 3 |
| 381 | CARRIER | F | 2022 | 2025 | 3 |
| 382 | CARRIER | F | 2022 | 2025 | 3 |
| 383 | CLEAR | F | 2022 | 2025 | 3 |
| 384 | CARRIER | M | 2023 | 2025 | 2 |
| 385 | CLEAR | F | 2022 | 2025 | 3 |
| 386 | CARRIER | F | 2022 | 2025 | 3 |
| 387 | CLEAR | F | 2022 | 2025 | 3 |
| 388 | CARRIER | F | 2022 | 2025 | 3 |
| 389 | CLEAR | F | 2022 | 2025 | 3 |
| 390 | CARRIER | F | 2022 | 2025 | 3 |
| 391 | CLEAR | M | 2023 | 2025 | 2 |
| 392 | CARRIER | F | 2022 | 2025 | 3 |
| 393 | CARRIER | F | 2022 | 2025 | 3 |
| 394 | CARRIER | F | 2022 | 2025 | 3 |
| 395 | CLEAR | F | 2022 | 2025 | 3 |
| 396 | CLEAR | F | 2022 | 2025 | 3 |
| 397 | CLEAR | F | 2023 | 2025 | 2 |
| 398 | CARRIER | F | 2023 | 2025 | 2 |
| 399 | CLEAR | M | 2023 | 2025 | 2 |
| 400 | CARRIER | F | 2023 | 2025 | 2 |
| 401 | CLEAR | M | 2023 | 2025 | 2 |
| 402 | AFFECTED | F | 2023 | 2025 | 2 |
| 403 | CLEAR | F | 2023 | 2025 | 2 |
| 404 | CARRIER | F | 2023 | 2025 | 2 |
| 405 | CARRIER | F | 2023 | 2025 | 2 |
| 406 | CLEAR | M | 2023 | 2025 | 2 |
| 407 | CARRIER | F | 2023 | 2025 | 2 |
| 408 | CARRIER | F | 2023 | 2025 | 2 |
| 409 | CARRIER | M | 2023 | 2025 | 2 |
| 410 | CLEAR | M | 2023 | 2025 | 2 |
| 411 | AFFECTED | F | 2023 | 2025 | 2 |
| 412 | CLEAR | F | 2023 | 2025 | 2 |
| 413 | CLEAR | F | 2023 | 2025 | 2 |
| 414 | CLEAR | F | 2023 | 2025 | 2 |
| 415 | CLEAR | F | 2023 | 2025 | 2 |
| 416 | CLEAR | F | 2023 | 2025 | 2 |
| 417 | AFFECTED | M | 2023 | 2025 | 2 |
| 418 | CARRIER | M | 2023 | 2025 | 2 |
| 419 | CLEAR | F | 2023 | 2025 | 2 |
| 420 | CLEAR | F | 2023 | 2025 | 2 |
| 421 | CARRIER | M | 2023 | 2025 | 2 |
| 422 | CARRIER | F | 2023 | 2025 | 2 |
| 423 | CLEAR | F | 2023 | 2025 | 2 |
| 424 | CARRIER | F | 2023 | 2025 | 2 |
| 425 | CLEAR | F | 2023 | 2025 | 2 |
| 426 | CARRIER | F | 2023 | 2025 | 2 |
| 427 | CLEAR | F | 2023 | 2025 | 2 |
| 428 | CLEAR | F | 2023 | 2025 | 2 |
| 429 | AFFECTED | F | 2023 | 2025 | 2 |
| 430 | CARRIER | M | 2024 | 2025 | 1 |
| 431 | CLEAR | M | 2024 | 2025 | 1 |
| 432 | CLEAR | M | 2024 | 2025 | 1 |
| 433 | CLEAR | M | 2024 | 2025 | 1 |
| 434 | CLEAR | F | 2023 | 2025 | 2 |
| 435 | CLEAR | F | 2023 | 2025 | 2 |
| 436 | AFFECTED | M | 2024 | 2025 | 1 |
| 437 | CLEAR | F | 2024 | 2025 | 1 |
| 438 | AFFECTED | M | 2024 | 2025 | 1 |
| 439 | CLEAR | M | 2024 | 2025 | 1 |
| 440 | CLEAR | F | 2024 | 2025 | 1 |
| 441 | AFFECTED | M | 2024 | 2025 | 1 |
| 442 | CLEAR | F | 2024 | 2025 | 1 |
| 443 | CARRIER | F | 2024 | 2025 | 1 |
| 444 | CLEAR | F | 2024 | 2025 | 1 |
| 445 | CLEAR | F | 2024 | 2025 | 1 |
| 446 | CLEAR | F | 2024 | 2025 | 1 |
| 447 | CLEAR | F | 2024 | 2025 | 1 |
| 448 | CARRIER | F | 2024 | 2025 | 1 |
| 449 | CARRIER | M | 2024 | 2025 | 1 |
| 450 | CARRIER | M | 2024 | 2025 | 1 |
| 451 | CLEAR | F | 2024 | 2025 | 1 |
| 452 | CLEAR | F | 2024 | 2025 | 1 |
| 453 | CLEAR | M | 2024 | 2025 | 1 |
| 454 | CLEAR | M | 2025 | 2025 | 0 |
| 455 | CARRIER | M | 2025 | 2025 | 0 |
| 456 | CARRIER | M | 2025 | 2025 | 0 |
| 457 | CLEAR | M | 2025 | 2025 | 0 |
| 458 | AFFECTED | M | 2025 | 2025 | 0 |
| 459 | CARRIER | M | 2025 | 2025 | 0 |
| 460 | CLEAR | M | 2025 | 2025 | 0 |
| 461 | CLEAR | M | 2025 | 2025 | 0 |
| 462 | CLEAR | F | 2025 | 2025 | 0 |
| 463 | CLEAR | M | 2025 | 2025 | 0 |
