# Supplementary material for: Prevalence and genotypic distribution of non-epidermolytic ichthyosis in Italian Golden Retrievers
Source: PLoS One. 2026 Mar 24;21(3):e0345595. doi: 10.1371/journal.pone.0345595 (PMC13012511; doi:10.1371/journal.pone.0345595)
Supplement: S2 Table — The table includes ABHD5 genotype results, PNPLA1 genotype (when available), sex, year of birth, year of sampling, and age at sampling for all tested dogs. (DOCX) [file pone.0345595.s002.docx]

**S2 Table. Individual-level data for ABHD5 genotyping in Italian Golden Retrievers.** The table includes ABHD5 genotype results, PNPLA1 genotype (when available), sex, year of birth, year of sampling, and age at sampling for all tested dogs.

| **ID** | **PNPLA1** | **ABHD5** | **sex** | **Year of birth** | **Year of Sampling** | **Age** |
| --- | --- | --- | --- | --- | --- | --- |
| 229 | CARRIER | CLEAR | M | 2017 | 2024 | 7 |
| 284 | AFFECTED | CLEAR | F | 2022 | 2024 | 2 |
| 376 | CLEAR | CLEAR | F | 2022 | 2025 | 3 |
| 464 | - | CLEAR | F | 2020 | 2025 | 5 |
| 465 | - | CLEAR | F | 2020 | 2025 | 5 |
| 466 | - | CLEAR | F | 2021 | 2025 | 4 |
| 467 | - | CLEAR | F | 2021 | 2025 | 4 |
| 468 | - | CLEAR | M | 2021 | 2025 | 4 |
| 469 | - | CLEAR | F | 2021 | 2025 | 4 |
| 470 | - | CLEAR | F | 2021 | 2025 | 4 |
| 471 | - | CLEAR | F | 2021 | 2025 | 4 |
| 472 | - | CLEAR | F | 2022 | 2025 | 3 |
| 473 | - | CLEAR | F | 2022 | 2025 | 3 |
| 474 | - | CLEAR | F | 2022 | 2025 | 3 |
| 475 | - | CLEAR | F | 2022 | 2025 | 3 |
| 476 | - | CLEAR | F | 2022 | 2025 | 3 |
| 477 | - | CLEAR | M | 2022 | 2025 | 3 |
| 478 | - | CLEAR | F | 2022 | 2025 | 3 |
| 479 | - | CLEAR | F | 2022 | 2025 | 3 |
| 480 | - | CLEAR | M | 2022 | 2025 | 3 |
| 481 | - | CLEAR | F | 2022 | 2025 | 3 |
| 482 | - | CLEAR | F | 2023 | 2025 | 2 |
| 483 | - | CLEAR | M | 2022 | 2025 | 3 |
| 484 | - | CLEAR | M | 2022 | 2025 | 3 |
| 485 | - | CLEAR | M | 2022 | 2025 | 3 |
| 486 | - | CLEAR | F | 2023 | 2025 | 2 |
| 487 | - | CLEAR | F | 2023 | 2025 | 2 |
| 488 | - | CLEAR | M | 2023 | 2025 | 2 |
| 489 | - | CLEAR | M | 2023 | 2025 | 2 |
| 490 | - | CLEAR | F | 2023 | 2025 | 2 |
| 491 | - | CLEAR | M | 2023 | 2025 | 2 |
| 492 | - | CLEAR | F | 2023 | 2025 | 2 |
| 493 | - | CLEAR | M | 2023 | 2025 | 2 |
| 494 | - | CLEAR | M | 2023 | 2025 | 2 |
| 495 | - | CLEAR | M | 2023 | 2025 | 2 |
| 496 | - | CLEAR | M | 2023 | 2025 | 2 |
| 497 | - | CLEAR | F | 2023 | 2024 | 1 |
| 498 | - | CLEAR | M | 2023 | 2025 | 2 |
| 499 | - | CLEAR | F | 2023 | 2025 | 2 |
| 500 | - | CLEAR | M | 2023 | 2025 | 2 |
| 501 | - | CLEAR | M | 2024 | 2025 | 1 |
| 502 | - | CLEAR | M | 2024 | 2025 | 1 |
| 503 | - | CLEAR | M | 2024 | 2025 | 1 |
| 504 | - | CLEAR | M | 2024 | 2025 | 1 |
| 505 | - | CLEAR | F | 2024 | 2025 | 1 |
