# Supplementary material for: Prevalence and genotypic distribution of non-epidermolytic ichthyosis in Italian Golden Retrievers
Source: PLoS One. 2026 Mar 24;21(3):e0345595. doi: 10.1371/journal.pone.0345595 (PMC13012511; doi:10.1371/journal.pone.0345595)
Supplement: S3 Table — The table reports the number and frequency of dogs classified as clear, affected, and carrier, stratified by sex. (DOCX) [file pone.0345595.s003.docx]

**S3 Table. *PNPLA1* genotype frequencies by sex**. The table reports the number and frequency of dogs classified as clear, affected, and carrier, stratified by sex.

| Sex | N° of  samples | Clears | | Affected | | Carriers | |
| --- | --- | --- | --- | --- | --- | --- | --- |
|  |  | N° | Freq | N° | Freq | N° | Freq |
| Female | 301 | 123 | 41% | 59 | 20% | 119 | 40% |
| Male | 162 | 69 | 43% | 39 | 24% | 54 | 33% |

N°= Number of samples

Freq= Frequencies
