# Supplementary material for: Prevalence and genotypic distribution of non-epidermolytic ichthyosis in Italian Golden Retrievers
Source: PLoS One. 2026 Mar 24;21(3):e0345595. doi: 10.1371/journal.pone.0345595 (PMC13012511; doi:10.1371/journal.pone.0345595)
Supplement: S4 Table — The table reports the number and frequency of dogs classified as clear, affected, and carrier, stratified by age class. (DOCX) [file pone.0345595.s004.docx]

**S4 Table. *PNPLA1* genotype frequencies by age**. The table reports the number and frequency of dogs classified as clear, affected, and carrier, stratified by age class.

| Age | N° of  samples | Clears | | Affected | | Carriers | |
| --- | --- | --- | --- | --- | --- | --- | --- |
|  |  | N° | Freq | N° | Freq | N° | Freq |
| 0 | 56 | 21 | 38% | 16 | 29% | 19 | 34% |
| 1 | 104 | 46 | 45% | 18 | 17% | 40 | 38% |
| 2 | 137 | 66 | 48% | 23 | 17% | 48 | 35% |
| 3 | 73 | 31 | 42% | 15 | 21% | 27 | 37% |
| 4 | 45 | 12 | 27% | 12 | 27% | 21 | 47% |
| 5 | 26 | 8 | 31% | 7 | 27% | 11 | 42% |
| 6-11 | 22 | 8 | 36% | 7 | 32% | 7 | 32% |

N°= Number of samples

Freq= Frequencies
